# Structure-Based Virtual Screening: Identification of a Novel NS2B-NS3 Protease Inhibitor with Potent Antiviral Activity against Zika and Dengue Viruses

**DOI:** 10.3390/microorganisms9030545

**Published:** 2021-03-06

**Authors:** Hye Jin Shin, Mi-Hwa Kim, Joo-Youn Lee, Insu Hwang, Gun Young Yoon, Hae Soo Kim, Young-Chan Kwon, Dae-Gyun Ahn, Kyun-Do Kim, Bum-Tae Kim, Seong-Jun Kim, Chonsaeng Kim

**Affiliations:** 1 Center for Convergent Research of Emerging Virus Infection, Korea Research Institute of Chemical Technology, Daejeon 34114, Korea; shinhy@krict.re.kr (H.J.S.); mihwa121215@gmail.com (M.-H.K.); bioseal2000@gmail.com (I.H.); emdoc96@krict.re.kr (G.Y.Y.); haiskim@krict.re.kr (H.S.K.); yckwon@krict.re.kr (Y.-C.K.); dgahn@krict.re.kr (D.-G.A.); kdkim@krict.re.kr (K.-D.K.); btkim@krict.re.kr (B.-T.K.); 2 Bioenvironmental Science and Toxicology Division, Gyeongnam Branch Institute, Korea Institute of Toxicology, Jinju 52834, Korea; 3 Therapeutics and Biotechnology Division, Korea Research Institute of Chemical Technology, 141 Gajeong-ro, Yuseong-gu, Daejeon 34114, Korea; leejy@krict.re.kr

**Keywords:** Zika virus, NS2B-NS3 protease, inhibitor, virtual screening

## Abstract

Zika virus (ZIKV), which is associated with severe diseases in humans, has spread rapidly and globally since its emergence. ZIKV and dengue virus (DENV) are closely related, and antibody-dependent enhancement (ADE) of infection between cocirculating ZIKV and DENV may exacerbate disease. Despite these serious threats, there are currently no approved antiviral drugs against ZIKV and DENV. The NS2B-NS3 viral protease is an attractive antiviral target because it plays a pivotal role in polyprotein cleavage, which is required for viral replication. Thus, we sought to identify novel inhibitors of the NS2B-NS3 protease. To that aim, we performed structure-based virtual screening using 467,000 structurally diverse chemical compounds. Then, a fluorescence-based protease inhibition assay was used to test whether the selected candidates inhibited ZIKV protease activity. Among the 123 candidate inhibitors selected from virtual screening, compound 1 significantly inhibited ZIKV NS2B-NS3 protease activity in vitro. In addition, compound 1 effectively inhibited ZIKV and DENV infection of human cells. Molecular docking analysis suggested that compound 1 binds to the NS2B-NS3 protease of ZIKV and DENV. Thus, compound 1 could be used as a new therapeutic option for the development of more potent antiviral drugs against both ZIKV and DENV, reducing the risks of ADE.

## 1. Introduction

Zika virus (ZIKV) and dengue virus (DENV) are closely related mosquito-borne single-stranded positive-polarity RNA viruses of the genus flavivirus in the *Flaviviridae* family [1]. ZIKV was first identified in a rhesus monkey in the Zika Forest region of Uganda in 1947 and has recently emerged in Brazil, rapidly spreading to other countries in South and North America in early 2015 [2,3,4]. ZIKV infection has been associated with neurological disorders such as Guillain-Barré syndrome in adults and microcephaly in newborns. DENV is easily transmitted between people by *Aedes aegypti* mosquitoes and has spread to more than 100 countries in Africa, the Americas, Asia, Pacific, and the Caribbean [5,6]. The four distinct serotypes of DENV have been reported to cause dengue fever (DF) and dengue shock syndrome (DSS). A primary DENV infection causes mild symptoms by inducing an immune response and protects from secondary infection by the same serotype. However, subsequent infection with a different serotype causes antibody-dependent enhancement (ADE) of infection due to preexisting antibodies, which are considered a risk factor for the development of DF/DSS [7]. ADE between cocirculating ZIKV and DENV has been reported [8,9,10], which could exacerbate the diseases caused by these viruses. Moreover, global warming and environmental destruction may cause the expansion of the habitat and populations of mosquitoes, thus directly contributing to the spread of ZIKV and DENV. Despite these serious threats, there are currently no approved antiviral drugs against ZIKV and DENV. Therefore, effective antiviral compounds are urgently needed to prevent or treat ZIKV and DENV infection.

After ZIKV and DENV infection of the host cell, the viral RNA is translated into a long polyprotein that is cleaved by a viral protease to generate three structural proteins (capsid, pre-membrane, and envelope proteins) and several nonstructural proteins (NS1, NS2A, NS2B, NS3, NS4A, NS4B, and NS5) that contribute to the formation of viral particles and replication of the viral genome [11,12]. The protease is an essential enzyme for viral replication and an attractive target for antiviral drug development [13,14]. For the treatment of human immunodeficiency virus (HIV) and hepatitis C virus (HCV) infection, nineteen protease inhibitor drugs are approved by the Food and Drug Administration (FDA) [15]. Both nonstructural proteins NS2B and NS3 are components of the ZIKV and DENV proteases, which are promising antiviral targets for the inhibition of viral replication, as protease inhibition could block the cleavage of the viral precursor [13,16].

Thus, we sought to identify novel protease inhibitors that could be used for the development of antiviral drugs. To that aim, we performed structure-based virtual screening targeting the binding site of the ZIKV NS2B-NS3 protease. More than 467,000 chemical compounds from the Korea Chemical Bank at the Korea Research Institute of Chemical Technology (KRICT) were screened to identify novel chemical inhibitors of the ZIKV NS2B-NS3 protease. To test whether the selected 123 candidates from the virtual screening inhibited ZIKV NS2B-NS3 protease in vitro, a fluorescence-based protease inhibition assay was performed. Compound 1 strongly inhibited ZIKV NS2B-NS3 protease activity. We determined that the addition of compound 1 effectively inhibits viral replication and reduces viral protein expression during in vitro ZIKV infection of human cells. Moreover, we found that compound 1 has potent antiviral activity against the closely related DENV. Thus, compound 1 could be used for the development of more potent antiviral drugs against ZIKV and DENV.

## 2. Materials and Methods

### 2.1. Cells, Viruses, and Compounds

HEK-293 cells (CRL-1573) were purchased from the American Type Culture Collection (ATCC). Huh7 cells were obtained from the JCRB Cell Bank. These cells were maintained in Dulbecco’s modified Eagle medium supplemented with 10% fetal bovine serum (both from HyClone, San Angelo, TX, USA) at 37 °C with 5% CO_2_ in a humidified incubator. The ZIKV strains PRVABC59 (ATCC VR-1843) and MR766 (ATCC VR-84) were purchased from ATCC. The ZIKV strain H/PF/2013 (001v-EVA1545) was obtained from the European Virus Archive. The ZIKV strain Asian (NCCP43245) and the DENV-4 strain (NCCP43257) were kindly provided by the Korean Centers for Disease Control and Prevention. A chemical library composed of 467,000 structurally diverse chemical compounds was obtained from the Korea Chemical Bank at the KRICT.

### 2.2. Structure-Based Virtual Screening for the Identification of ZIKV NS2B-NS3 Protease Inhibitors

Structure-based virtual screening was carried out using the Schrodinger Suite v.2018-1 (Schrödinger, LLC, New York, NY, USA, 2018). The X-ray crystal structure of the NS2B-NS3 protease from ZIKV in complex with EN300 ((1H-benzo[d]imidazol-1-yl)methanol) was obtained from the Protein Data Bank (PDB code 5H4I) [17]. The protein structure was revised using Protein Preparation Wizard in Maestro v.11.5, and a cubic receptor grid box with 30 Å × 30 Å × 30 Å centered on the complexed ligand was generated. The 467,650-member Korea Chemical Bank compound library was subjected to ligand preparation using the LigPrep v.4.4 applying the OPLS_2005 force field. During the process, tautomer and ionization states at pH 7.0 ± 2.0 were generated using the Epik v.4.3 module. Compound docking was performed using the Glide v.7.8 program (Schrodinger, New York, NY, USA) with SP (Standard Precision) mode. Based on visual inspection, 123 compounds were selected for the in vitro protease inhibition assay.

### 2.3. In Vitro ZIKV Protease Inhibition Aassay

The recombinant ZIKV NS2B-NS3 protease was produced as described elsewhere with a brief modification [13]. The codon-optimized coding sequence of ZIKV protease was synthesized by Integrated DNA Technologies and was inserted into the pET22b vector. This plasmid was transformed into *E. coli* BL21(DE3) and induced with IPTG. His-tag fused protease was purified using Ni-NTA affinity column (Qiagen, Dusseldorf, Germany). Purified protein was dialyzed against PBS and analyzed by Coomassie blue staining. To detect ZIKV NS2B-NS3 protease activity, a fluorescence-based protease assay was performed as described previously [18]. The substrate benzoyl-norleucine-lysine-lysine-arginine 7-amino-4-methylcoumarine (Bz-Nle-K-K-R-AMC) was purchased from PEPTRON, Republic of Korea, and dissolved in DMSO. The ZIKV protease was mixed with indicated concentrations of inhibitors and initiated the cleavage reaction by the addition of substrate. After incubation for 1 h at 37 °C, the fluorescence signal was measured at 460 nm with excitation at 360 nm using a Synergy H1 multi-mode microplate reader (BioTek). The protease activity after each treatment was calculated as a relative percentage to the control (DMSO control = 100%, no protease control = 0%).

### 2.4. Cell Viability Assay

The 50% effective cytotoxic concentration (CC_50_) value of compound 1 was determined in Huh7 cells using the thiazolyl blue tetrazolium bromide (MTT, Sigma-Aldrich, St. Louis, MO, USA) assay. Cells were seeded in 96-well plates and treated with the compound, which was serially diluted 5-fold from 50 µM to 0.4 µM. After 72 h of treatment at 37 °C, MTT (5 mg/mL in PBS solution) was diluted to a 1:3 ratio and added to each well. The plate was incubated at 37 °C for 1 h and placed on a shaker with MTT solvent to dissolve the dye. Cell viability was calculated as a percentage relative to that of DMSO-treated control cells (defined as 100%).

### 2.5. Western Blotting and Real Time RT-PCR

HEK293 cells were seeded in 12-well plates (1 × 10^5^ cells/well) and simultaneously treated with compound 1 and ZIKV at a multiplicity of infection (MOI) of 1. Total cell lysates were harvested and analyzed using real-time RT-PCR and Western blotting at 72 h post-infection. For RT-PCR, total cellular RNA was purified using the QIAGEN RNeasy Mini kit according to the manufacturer’s instructions. Reverse transcription was performed using the ZIKV forward (5′-GGA TGG TGC AAA GGG AAG GC-3′) and ZIKV reverse (5′-GGG GGA GTC AGG ATG GTA CT-3’) primers to measure the amount of viral RNA. β-actin mRNA was used as a loading control with forward (5′-GAT GCA GAA GGA GAT CAC TG-3′) and reverse (5′-CTG CTT GCT GAT CCA CAT-3′) primers. For Western blotting, the ZIKV envelope antibody (GeneTex, GTX133314, Irvine, CA, USA), ZIKV NS3 antibody (GeneTex, GTX133320), and β-actin antibody (Sigma-Aldrich, A1978, St. Louis, MO, USA) were used.

### 2.6. Immunofluorescence Microscopy

Huh7 cells were plated in 96-well plates at 2 × 10^4^ cells/well and infected with ZIKV or DENV at a MOI of 2. Cells were simultaneously treated with 5-fold serial dilutions (from 25 µM to 0.2 µM) of compound 1 and incubated for 72 h. For immunofluorescence, cells were fixed and permeabilized with a 3:1 mixture of ice-cold methanol-acetone. The primary antibody to dsRNA (English Scientific Consulting) and anti-mouse Alexa Fluor 488-conjugated secondary antibody (Life Technologies, #A11001, Carlsbad, CA, USA) were diluted in PBS and incubated for 1 h at room temperature, followed by counterstaining with Hoechst 33342 (Life Technologies, #H3572). Images were captured using an Operetta system (Perkin Elmer, Waltham, MA, USA). The HARMONY software in the Operetta system was used to quantify the ratio of infected cells to total cells.

### 2.7. Molecular Docking for the DENV NS2B-NS3 Protease

We performed molecular docking using the Schrodinger Suite v.2019-1 (Schrödinger, LLC, New York, NY, USA, 2019) to predict the binding model of compound 1 to the DENV-4 NS2B-NS3 protease. The X-ray structure of the DENV-4 NS2B-NS3 protease (PDB code 5YW1) was obtained from the Protein Data Bank. Protein preparation was revised using Protein Preparation Wizard in Maestro v.11.9. A receptor grid box for docking of 30 Å × 30 Å × 30 Å in size centered on the complexed peptide at the binding site was generated. The ligand was minimized using an OPLS_2005 force field with a dielectric constant of 80.0 in MacroModel v.12.3 (Schrodinger, New York, NY, USA). Molecular docking of the ligand was performed using the SP mode in Glide v.8.2 (Schrodinger, New York, NY, USA). The predicted binding models of the ligand for the ZIKV and DENV-4 NS2B-NS3 proteases were represented using Discovery Studio 2018 (Dassault Systèmes BIOVIA, San Diego, CA, USA, 2018).

## 3. Results

### 3.1. Identification of a ZIKV NS2B-NS3 Protease Inhibitor by Virtual Screening

**Figure 1:**
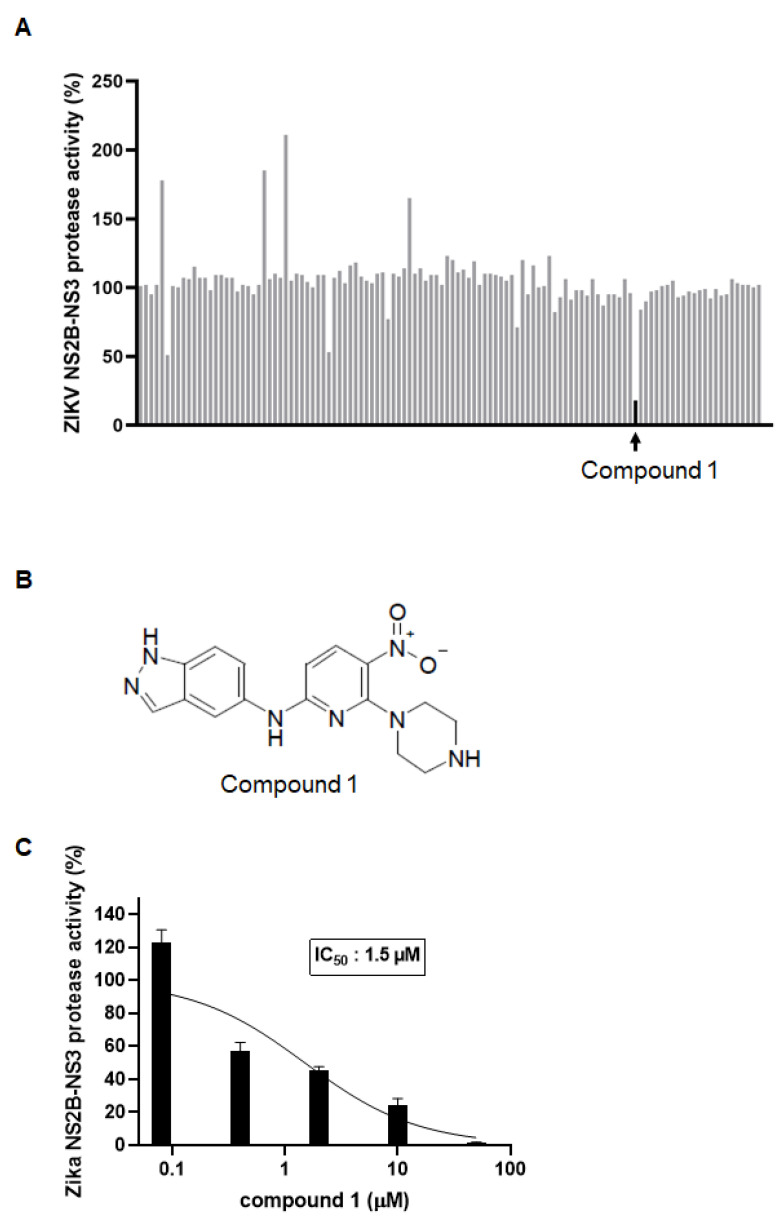
Identification of a novel inhibitor of the Zika virus (ZIKV) NS2B-NS3 protease using structure-based virtual screening and an in vitro protease inhibition assay. (**A**) The ZIKV NS2B-NS3 protease and substrate were mixed with 123 candidate compounds (50 µM) selected from virtual screening. After incubation for 1 h at 37 °C, the fluorescent signal was measured using a plate reader. The protease activity after each treatment was calculated as a relative percentage to the control (DMSO control was set as 100%, no protease control was set as 0%). (**B**) Chemical structure of compound 1. (**C**) The ZIKV NS2B-NS3 protease and substrate were mixed with the indicated concentrations of compound 1, and the fluorescence-based protease inhibition assay was performed. Protease inhibition activity (IC_50_) was determined from a dose-response curve.

Based on the ZIKV NS2B-NS3 protein structure (PDB code 5H4I), we performed structure-based virtual screening targeting the binding site of the protease using a chemical compound library to identify novel inhibitors of the ZIKV protease. The chemical compound library was composed of 467,000 structurally diverse chemical compounds that were obtained from the Korea Chemical Bank (Korea Research Institute of Chemical Technology, Republic of Korea). Small-molecule inhibitor candidates showing an inhibitory interaction with the NS2B-NS3 protease active site were selected using primary structure-based screening. High-throughput virtual screening (HTVS) selected 123 compounds with docking scores and visual inspection of the ligand-receptor binding mode for structural diversity. To test whether the selected 123 candidates from the virtual screening inhibited ZIKV protease activity, a fluorescence-based protease inhibition assay was performed. The fluorescence signal from the substrate cleaved by the protease decreased upon treatment with the inhibitory compounds. Compared with the control (DMSO), compound 1 showed the most effective protease activity inhibition in vitro (Figure 1A). The chemical structure of compound 1 is depicted in Figure 1B. To determine the half-maximal inhibitory concentration (IC_50_), compound 1 serially diluted 5-fold from 50 µM to 0.08 µM was treated with the protease and substrate. As shown in Figure 1C, compound 1 inhibited ZIKV NS2B-NS3 protease activity in a dose-dependent manner, with an apparent IC_50_ of 1.5 µM. Overall, a novel inhibitor of ZIKV NS2B-NS3 was identified using structure-based virtual screening and an in vitro protease inhibition assay.

### 3.2. Compound 1 Potently Inhibits ZIKV Infection of Human Cells

**Figure 2:**
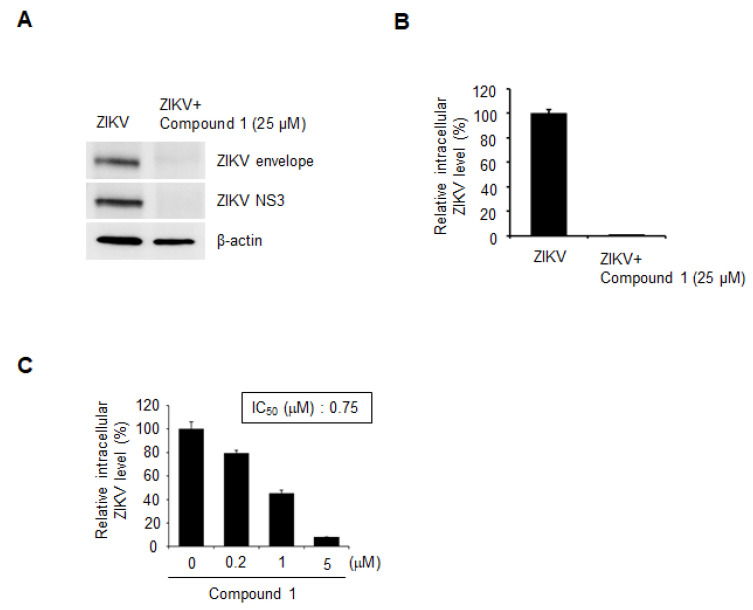
Compound 1 potently inhibits ZIKV infection of HEK-293 cells. (**A**) HEK-293 cells were simultaneously incubated with ZIKV (strain: PRVABC59) and compound 1 (25 µM). After 72 h, cell extracts were prepared from the cells and subjected to Western blot analysis with anti-ZIKV envelope and anti-ZIKV NS3 antibodies. β-actin was used as a loading control. (**B**) Cellular RNAs were prepared from cells in (**A**) and subjected to real-time RT-PCR for ZIKV RNA. (**C**) Cellular RNAs were isolated from cells infected with ZIKV and treated with the indicated concentrations of compound 1. The level of ZIKV RNA was measured by real-time RT-PCR. Antiviral activity (IC_50_) was determined from a dose-response curve.

To examine whether compound 1 potently inhibits ZIKV infection of human cells, HEK-293 cells were infected with ZIKV (strain: PRVABC59) and immediately treated with 25 µM of compound 1. At 72 h post-infection, total cell lysates were prepared and analyzed by Western blotting with anti-ZIKV envelope and NS3 antibodies. The ZIKV envelope and NS3 proteins were hardly detected upon treatment with compound 1 (Figure 2A). To test whether compound 1 could inhibit ZIKV replication, viral RNA was measured using real-time RT-PCR. Treatment of ZIKV-infected cells with 25 µM of compound 1 dramatically reduced the amount of ZIKV RNA (Figure 2B). Increasing concentrations of compound 1 (0.2–5 µM) were added to ZIKV-infected cells. As shown in Figure 2C, compound 1 inhibited ZIKV RNA replication in a dose-dependent manner, with an IC_50_ of 0.75 µM. These results demonstrated that compound 1 is a potent ZIKV inhibitor, with IC_50_ values in the sub-micromolar range.

To further confirm the antiviral activity of compound 1, Huh7 cells were infected with ZIKV (strain: PRVABC59) and then treated with 5-fold serial dilutions (25–0.2 µM) of compound 1 for 72 h. For the immunofluorescence assay, ZIKV-infected cells were stained with an anti-dsRNA antibody to detect viral dsRNA. ZIKV-infected Huh7 cells were visualized using a high-content imaging system and quantified by counting the number of cells positive for dsRNA. The inhibitory activity of each treatment was calculated as the percentage of positive cells relative to that in DMSO-treated control cells.

**Figure 3:**
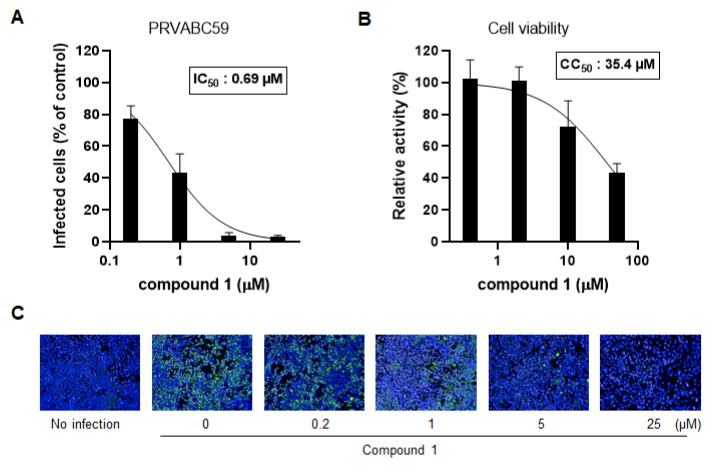
Compound 1 potently inhibits ZIKV infection of Huh7 cells. (**A**) Huh7 cells were treated with increasing concentrations of compound 1 and infected with ZIKV (strain: PRVABC59). After 72 h, infected cells were stained with an anti-dsRNA primary antibody and an Alexa Fluor 488-conjugated secondary antibody. Nuclei were counterstained with Hoechst 33342. Viral infection was calculated by counting the stained cells and the antiviral activity (IC_50_) was determined from a dose-response curve. (**B**) Huh7 cells were treated with the indicated concentrations of compound 1 without ZIKV infection and analyzed for cell viability using the thiazolyl blue tetrazolium bromide (MTT) assay. (**C**) Representative images of (**A**).

Compound 1 potently inhibited ZIKV infection in a dose-dependent manner, with an IC_50_ of 0.69 µM (Figure 3A,C). In addition, we treated Huh7 cells with a broad range of compound 1 concentrations (0.4 µM–50 µM) to analyze its cytotoxic effect using the MTT assay. Compound 1 showed mild cytotoxicity in a dose-dependent manner, with a CC_50_ of 35.4 µM (Figure 3B). The ratio of the toxic and effective doses indicates the selectivity index, which quantifies the relative safety of a drug. The selectivity index for compound 1 was 51.3.

**Figure 4:**
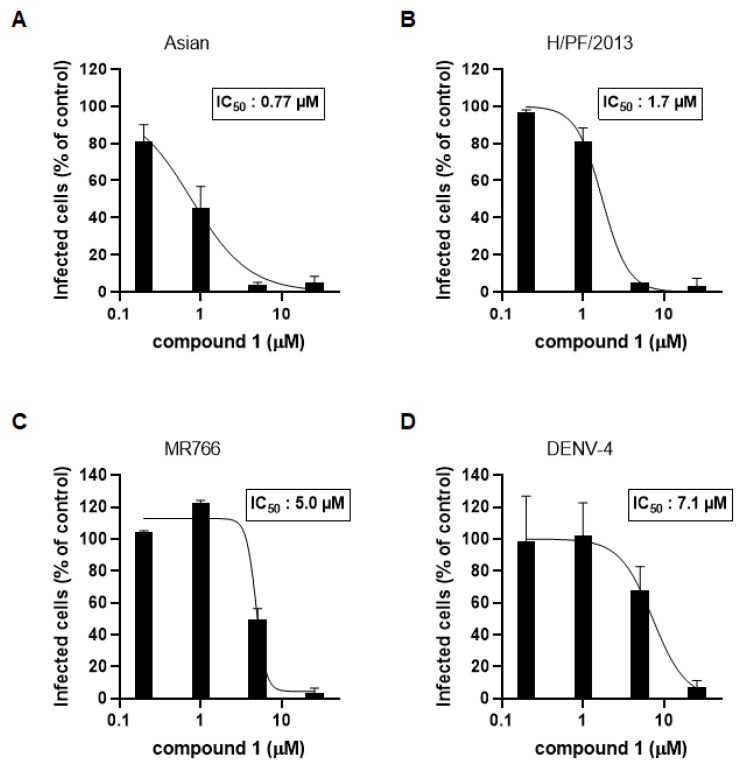
Compound 1 potently inhibits the infection of Huh7 cells by diverse strains of ZIKV and dengue virus type 4 (DENV-4, NCCP43257). Huh7 cells were treated with increasing concentrations of compound 1 and infected with ZIKV (strain: Asian) (**A**), (strain: H/PF/2013) (**B**), (strain: MR766) (**C**), and DENV-4 (**D**). A similar analysis to that shown in Figure 3A was performed.

We also tested whether compound 1 could inhibit the replication of three other ZIKV strains in Huh7 cells, including the Asian strain, the MR766 strain, and the H/PF/2013 strain. Increasing concentrations of compound 1 (0.2–25 µM) were used to treat ZIKV-infected cells for 72 h. We performed the immunofluorescence assay as described above for Figure 3. As shown in Figure 4A,B, compound 1 was effective at inhibiting infection of Huh7 cells by the Asian and H/PF/2013 strains, with IC_50_ values of 0.77 µM and 1.7 µM, respectively. These results also showed that the inhibition efficacy was similar to that against PRVABC59 (Figure 3A). For strain MR766, compound 1 had a slightly higher IC_50_ value than for other ZIKV strains (Figure 4C). Collectively, these results clearly demonstrated that compound 1 is a potent inhibitor of ZIKV.

### 3.3. Compound 1 Also Inhibits DENV Infection

ZIKV and DENV are closely related viruses. The NS2B-NS3 proteases of ZIKV and DENV share amino acid sequence identity, which results in a similar crystal structure. In order to determine whether compound 1 could also inhibit DENV infection, Huh7 cells were infected with DENV type 4 (DENV-4), and then treated with 5-fold serial dilutions (25–0.2 µM) of compound 1 for 72 h. An immunofluorescence assay was used to measure antiviral activity. Compound 1 inhibited DENV-4 infection, with an IC_50_ value of 7.1 µM (Figure 4D). Thus, compound 1 inhibited ZIKV and DENV infection of human cells.

### 3.4. Predicted Binding Modes of Compound 1 for Viral NS2B-NS3 Proteases

**Figure 5:**
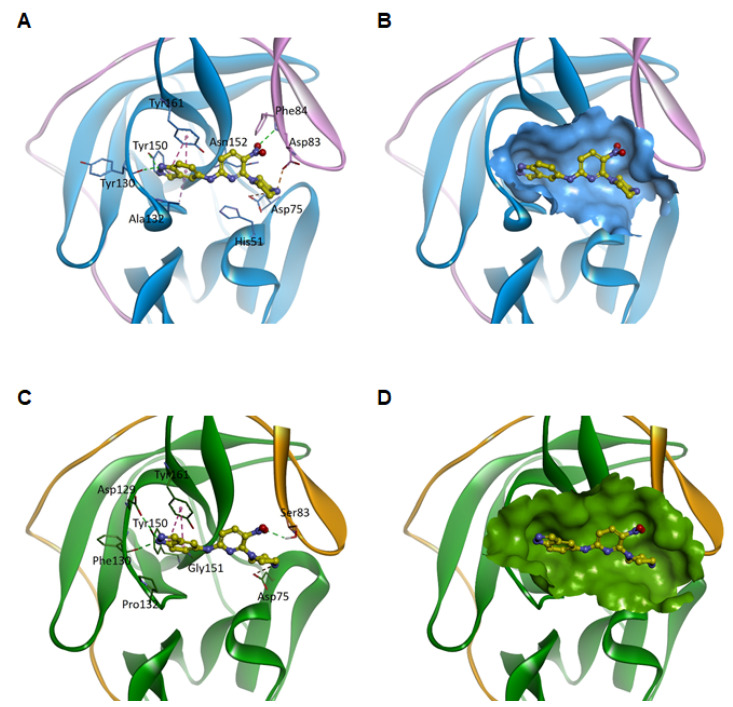
Predicted binding modes of compound 1 for viral NS2B-NS3 proteases. (**A**) Binding of compound 1 (yellow ball and stick model) to the ZIKV NS2B-NS3 protease (pink-blue ribbon model) and (**B**) surface model of the binding site of ZIKV NS2B-NS3. (**C**) Binding of compound 1 (yellow ball and stick model) to the DENV-4 NS2B-NS3 protease (orange-green ribbon model) and (**D**) surface model of the binding site of DENV-4 NS2B-NS3. For clarity, key binding site residues are shown in sticks and labeled using the 3-letter amino acid code. The hydrogen bonds are displayed as green dashed lines and hydrophobic interactions are shown as pink dashed lines. In addition, electrostatic interactions are indicated by orange dashed lines.

According to the virtual screening model using docking, compound 1 bound to the active site of the protease. The proposed binding mode of compound 1 to ZIKV NS2B-NS3 protease is shown in Figure 5A,B. In this model, the indazole ring was positioned slightly deeper in the cavity surrounded by the hydrophobic side chains of Ala132, Tyr150, and Tyr161. Two nitrogen atoms of the indazole group interacted with the backbone carbonyl groups of Tyr130 and Tyr150 through hydrogen bonds. In addition, the nitro group formed a hydrogen bond with the backbone amide group of Phe84, and the nitrogen of piperazine formed electrostatic interactions with Asp75 and Asp83.

In addition, we performed molecular docking analysis to predict the binding mode of compound 1 to the DENV-4 NS2B-NS3 protease (PDB code 5YW1). The binding models are shown in Figure 5C,D. The proposed binding modes of compound 1 to the DENV-4 protease are very similar to those for the ZIKV protease. The indazole ring of compound 1 was bound to the pocket formed by the hydrophobic side chains of Pro132, Tyr150, and Tyr161. One nitrogen atom of the indazole ring forms hydrogen bonded with Asp129 and the backbone carbonyl group of Phe130. In addition, the nitro group formed a hydrogen bond with Ser83, and the nitrogen of piperazine formed electrostatic interactions with Asp75. Compared with ZIKV, we could not observe hydrogen bonding between the nitrogen of the indazole ring and Tyr150. These results suggest that the inhibitory activity of compound 1 on the DENV-4 NS2B-NS3 protease is lower than that on the ZIKV NS2B-NS3 protease.

## 4. Discussion

Despite the serious problems caused by ZIKV and DENV, there are currently no effective drugs against these viruses. To identify a novel antiviral agent against ZIKV, we used its protease as a target for inhibitor screening, owing to the crucial role of the protease in polyprotein processing and viral replication. In silico virtual screening based on the structure of the ZIKV NS2B-NS3 protease led us to identify 123 potential inhibitors from 467,000 compounds. The most potent inhibitor, compound 1, was selected from the 123 potential inhibitors using an in vitro protease inhibition assay. This compound showed antiviral activity against four ZIKV strains, with IC_50_ values in the sub-micromolar range. In addition, it inhibited infection by a DENV strain. We performed molecular docking analysis to predict the binding mode of compound 1 with the NS2B-NS3 protease of ZIKV and DENV.

Importantly, compound 1 inhibited the replication of ZIKV strains from two different geographical lineages, African and Asian. Since the recent worldwide outbreak of ZIKV, the Asian lineage continues to spread in the Americas and other countries of the world [19]. From our results, it seems that compound 1 may confer protection against a broad range of circulating strains. Therefore, we propose compound 1 as a potential candidate for the treatment of ZIKV infection over a broad geographic range.

Protease inhibitors have been developed as therapeutics for HIV and HCV [20,21]. Several studies have identified diverse inhibitors of the ZIKV protease [18,22,23,24]. By comparing the antiviral activity of these inhibitors, compound 1 showed better efficacy than the previously identified compounds at inhibiting the ZIKV NS2B-NS3 protease. Most of the previous studies have only reported the antiviral effects on ZIKV. In contrast, we also demonstrated the antiviral effect of compound 1 on DENV. ADE between ZIKV and DENV has been reported [8,10]. ZIKV has cocirculated with DENV; thus, ADE may exacerbate the diseases caused by these two viruses. Antiviral drugs that inhibit both viruses, such as compound 1, could help to reduce these threats. Two studies identified a novel series of 2,5,6-trisubstituted pyrazine compound as a potent allosteric inhibitor of ZIKV protease with IC_50_ value as low as 130 nM [25,26]. This compound potently inhibited ZIKV replication in cells with EC68 values of 300–600 nM in a mouse model of ZIKV infection. The activity of their compound was comparable with our compound 1 in the cell culture model. Rachel et al. identified the five-lipoxygenase-activating protein inhibitor, MK-591, inhibiting the ZIKV protease and infection in neural stem cells [27]. The IC_50_ was higher (3 µM) than our compound 1. A natural active compound derived from black tea, theaflavin-3,3′-digallate, with an IC_50_ of 2.3 µM was identified [28]. Asunaprevir and Simeprevir were identified using the pharmacophore anchor model to have potent anti-ZIKV activities with IC_50_ values 4.7 µM and 0.4 µM [11]. Szymon et al. identified small molecule inhibitors of ZIKV protease with a low micromolar affinity for protease [29]. Hani et al. identified seven compounds as potential inhibitors of ZIKV protease by virtual screening and molecular docking studies [30]. These inhibitors can be used for further experimental verification. The antihistaminic chlorcyclizine was identified as a ZIKV protease inhibitor by structure-based virtual screening and drug repurposing approaches [31]. The IC_50_ was 69.0 µM. Lucy et al. identified potential ZIKV protease inhibitors via virtual screening [32]. It is still necessary to test that these compounds inhibit the ZIKV infection. Temoporfin was identified as a potent ZIKV inhibitor with nanomolar potencies [33]. As this drug has been approved for clinical use, it could be used as a promising therapy for ZIKV infection. NSC135618 was identified as a broad spectrum flavivirus protease inhibitor [34]. This compound significantly reduced titers of ZIKV on A549 cells with low micromolar potency. Emricasan was identified as a potent inhibitor of ZIKV with the IC_50_ values of 0.13–0.9 µM against three ZIKV strains [35]. Further validation of this drug inhibiting the ZIKV protease is required.

Although further validation of these results in an animal model of ZIKV and DENV infection is required, compound 1 could be a new promising lead compound for the design of more potent antiviral drugs against ZIKV and DENV. 

## Data Availability

The data presented in this study are available within this article.
